# Advances and Future Applications of Augmented Peripheral Nerve Regeneration

**DOI:** 10.3390/ijms17091494

**Published:** 2016-09-07

**Authors:** Salazar Jones, Howard M. Eisenberg, Xiaofeng Jia

**Affiliations:** 1 Department of Neurosurgery, University of Maryland School of Medicine, Baltimore, MD 21201, USA; sjones2@smail.umaryland.edu (S.J.); heisenberg@smail.umaryland.edu (H.M.E.); 2 Department of Orthopaedics, University of Maryland School of Medicine, Baltimore, MD 21201, USA; 3 Department of Anatomy and Neurobiology, University of Maryland School of Medicine, Baltimore, MD 21201, USA; 4 Department of Biomedical Engineering, The Johns Hopkins University School of Medicine, Baltimore, MD 21205, USA; 5 Department of Anesthesiology and Critical Care Medicine, The Johns Hopkins University School of Medicine, Baltimore, MD 21205, USA

**Keywords:** peripheral nerve, peripheral nerve regeneration, stem cells, neurotrophic factors, nerve conduits

## Abstract

Peripheral nerve injuries remain a significant source of long lasting morbidity, disability, and economic costs. Much research continues to be performed in areas related to improving the surgical outcomes of peripheral nerve repair. In this review, the physiology of peripheral nerve regeneration and the multitude of efforts to improve surgical outcomes are discussed. Improvements in tissue engineering that have allowed for the use of synthetic conduits seeded with neurotrophic factors are highlighted. Selected pre-clinical and available clinical data using cell based methods such as Schwann cell, undifferentiated, and differentiated stem cell transplantation to guide and enhance peripheral nerve regeneration are presented. The limitations that still exist in the utility of neurotrophic factors and cell-based therapies are outlined. Strategies that are most promising for translation into the clinical arena are suggested.

## 1. Introduction

Peripheral nerve injuries remain a significant source of long lasting morbidity, disability, and economic costs. The associated neuropathic pain and disability are the leading determinants for impacted quality of life in peripheral nerve injuries [1]. Injuries to the peripheral nerves can occur in multiple clinical scenarios. From 1% to 3% of trauma patients will have an injury involving a peripheral nerve [2,3]. The most common associations are the male gender and motor vehicle accidents [1]. Nerve injuries have been recognized as occurring during falls and in children [4,5]. Iatrogenic peripheral nerve injury is seen in surgery, anesthesia injections, chemotherapy, and radiation for breast or head and neck tumors [6,7,8]. Obstetrical brachial plexus injury is seen in 1.24 per 1000 births [9]. Peripheral nerve injuries including the brachial plexus, radial, and ulnar nerves are significantly higher during wartime in all branches of the military [10]. During Operation Iraqi Freedom, 3% of all extremity injuries involved damage to peripheral nerves [11].

## 2. Pathogenesis of Nerve Injury and Repair

In contrast to the central nervous system, the peripheral nervous system has the ability to regenerate. There is some evidence that a conditioning lesion primes the peripheral nerve for regeneration [12]. However, functional recovery is often incomplete.

The regenerative process starts with the initial response to injury [13]. After nerve transection, an orchestration of morphologic changes occurs in the soma, proximal axon, and distal axonal stump. In the soma, there is dissolution of Nissl bodies and peripheral displacement of the nucleus [13]. The neuronal mRNA transcription profile changes after injury to support axon regeneration and neuronal survival [14]. Proteins associated with neuronal growth are upregulated such as GAP-43, tubulin, actin, and multiple neuropeptides and cytokines [15]. The proximal nerve stump retracts back to its node of Ranvier [16]. The distal nerve undergoes anterograde or Wallerian degeneration [17]. The proximal stump sprouts processes that sample the environment for neurotrophic factors to guide them to their target [18,19].

Schwann cells play an important role in the axonal regeneration. Schwann cells deposit laminin, fibronectin, tenascin, heparin sulfate, and collagen to bolster the extracellular matrix lost from injury [15]. Schwann cells express cell adhesion molecules that are important in interacting with matrix proteins that will modulate axon outgrowth and pathfinding [15,20,21]. Schwann cells produce chemokines to attract macrophages for the removal of myelin and axonal debris [22,23]. Schwann cells also elongate along their basal lamina in bands of Bungner to provide the scaffolding for new axonal growth [22,23].

In mixed nerves with both motor and sensory axons, regenerating motor and sensory axons should grow along the proper pathways to prevent haphazard regeneration. Intrinsic mechanisms are in place that allow motoneurons to preferentially regenerate along motor pathways even if the nerve graft is misaligned [24]. One pathway is through the small GTP-binding protein RhoA and effector Rho-kinase (ROCK) [25]. RhoA differentially affects motor and sensory axonal regeneration [25]. The RhoA/ROCK pathway is significant especially since the use of a ROCK inhibitor has been experimentally shown to facilitate growth of motoneurons over sensory neurons [25].

Regeneration is limited by the axonal growth rate commonly stated as 1 mm per day, though it can vary depending on location [26]. In the clinical setting, a positive Tinel’s sign elicited by neuropathic pain, reproduced by tapping over a nerve, can help localize the regenerating nerve front. However, in the absence of a migrating Tinel’s sign, the clinician is in the dark regarding nerve regeneration. Common practice is to allow several weeks or months to monitor for nerve recovery before proceeding with a nerve exploration. The senior author has previously worked on intrafascicular electrodes and has developed biodegradable regenerative type conduits that possess electrodes for the monitoring of nerve recovery [27,28,29,30]. Gamble et al. has also investigated implantable nerve stimulators that serve to monitor nerve regeneration [31]. Such methods will improve our temporal understanding of complete, incomplete, and failed nerve regeneration. In our current understanding, incomplete recovery is related to the time dependent neurologic and non-neurologic cellular responses to injury [22]. Nerve injuries with gaps are subject to the ingrowth of scar tissue. Large gaps affect the Schwann cell’s ability to form adequate bands of Bungner; hence, exogenous methods of scaffolds or conduits are used.

In chronic axotomy and chronic muscle denervation, inadequate neurotrophic factors from Schwann cells and target organs that diminish over time lead to poorer outcomes [32]. Untreated nerve injuries have several histological and molecular changes primarily in the distal stump, such as a decrease in Schwann cells and an increase in fibroblasts [33]. Jonsson et al. suggests three months as the critical time point where the effects of medical intervention start to decrease [33]. After that time period, there is a dramatic decrease in the number of regenerating motoneurons and myelinated axons, with greater fibrosis and scarring in the distal nerve stump [33]. The distal nerve stump degrades very quickly and loses its ability to reconnect with its previous axon as a function of time (as short as 4 weeks) and distance [34]. In the absence of reinnervation, the muscle undergoes fibrofatty infiltration [35].

## 3. Surgical Methods of Nerve Repair

In the clinical setting, there are various surgical techniques at the disposal of the peripheral nerve surgeon. For peripheral nerve injuries that do not involve a gap, tension-free primary neurorrhaphy is the method of repair. For a peripheral nerve injury with a gap that precludes a tension-free repair, bridging the gap with a nerve autograft remains the standard of care [19,36]. The sural nerve is the most common donor nerve [19]. However, this practice is associated with donor site morbidity [19]. The sural nerve, a sensory nerve, is not always an appropriate size match for grafting, hence the practice of cable grafting [19]. Repair of a nerve with mixed motor and sensory components is better with a motor or mixed nerve autograft, rather than a sensory autograft [37].

An alternative to nerve autografts is the use of autogenous or synthetic conduits [19,38,39,40,41]. Vein and composite muscle-in-vein conduits have been used in patients with digital nerve injuries with similar results to autografts [42]. In animal models, platelet-rich plasma within the vein conduit has been used to demonstrate improved functional and histological outcomes at 6 weeks [43]. Using veins as conduits has been described, but requires a donor site for harvesting.

Nerve allografts are another option for peripheral nerve repair that avoid the donor site morbidity seen with autografts. However, the immunogenicity of nerve allografts require concurrent administration of immunosuppressants for up to 18 months [44]. Protocols have been established that create acellular nerve allografts to reduce the immunogenicity of nerve allografts while preserving the extracellular matrix. Such protocols often involve detergent processing, freeze-thaw cycles, and cold preservation. The proprietary preparation of AxoGen (Alachua, FL, USA) allografts involve a combination of detergent processing, enzymatic digestion, and gamma irradiation [45]. Of particular note, the method of preparing the acellular nerve allograft can affect the overall regeneration. Detergent-processed allografts showed similar performance to autografts, whereas cold-preserved allografts and AxoGen allografts showed inferior nerve regeneration in comparison to autografts [45]. In a similar pre-clinical rat sciatic model, regeneration across gaps of 14 and 28 mm using AxoGen allografts were inferior to autografts [46].

Synthetic nerve conduits made of polyglycolic acid, polyester, collagen, or polycaprolactone are available as “off-the-shelf” products [40] (See Table 1). Of the Food and Drug Administration (FDA) approved nerve conduits, the collagen-based NeuraGen^®^ (Integra LifeSciences, Plainsboro, NJ, USA) and polyglycolic acid NeuroTube^®^ (Synovis Micro Companies Alliance, Birmingham, AL, USA) conduits have the most encouraging clinical data. Taras et al. showed good sensory recovery of 22 digital nerves across an average gap of 12 mm using NeuraGen^®^ [47]. Wangensteen et al. showed that NeuraGen^®^ is adaptable for use throughout the body but had modest nerve improvement in only 43% of patients in a heterogenous trauma population [48]. Ashley et al. evaluated the use of NeuraGen^®^ in five infants with brachial plexus injuries, with four of the five infants gaining significant motor improvement with the ability to feed and dress themselves at 2 years follow-up [49]. In a comparison of the NeuraGen^®^ to the AxoGen allograft, the NeuraGen^®^ produced inferior results in a rat model across 14 and 28 mm gaps [46]. Collagen tubules have been evaluated by other investigators. Boeckstyns et al. showed similar sensory and motor recovery at 24 months of ulnar and median nerve lacerations after collagen based conduit repair compared to neurorrhaphy for gaps 6 mm or less [50]. Kuffler et al. formed collagen tubules from sheets and filled them with platelet-rich fibrin to span across a 12 cm gap in a patient with an ulnar neuroma. Kuffler et al.’s patient showed initial motor recovery at 1.5 years and full motor recovery at 2 years.

The largest clinical series evaluating polyglycolic acid conduits was performed by Weber et al. [58]. Weber et al. demonstrated in 62 repaired digital nerves that nerve gaps less than 4 mm had better two-point discrimination when repaired with polyglycolic acid conduits versus primary neurorrhaphy [58]. Battiston et al.’s series of 17 patients with 19 digital nerve injuries repaired using NeuroTube^®^ conduits across gaps 1–4 cm showed similar positive results [39]. Significant motor recovery using NeuroTube^®^ was reported by Rosson et al.’s retrospective case series of six patients with various upper extremity nerve gaps 1.5–4 cm in length [51]. All six patients had greater than anti-gravity strength on follow-up [51]. A known disadvantage of the NeuroTube^®^ is the extrusion of the conduit, even through healthy appearing tissues [52]. Weber et al. also reported three cases of conduit extrusion in his series [58].

Pre-clinical data in rats with a 10 mm sciatic nerve gap using Neurolac^®^ suggested favorable motor recovery with the Neurolac^®^ comparable to autografts [59]. The clinical data on the use of the Neurolac^®^ has been less encouraging. Bertleff et al. used Neurolac^®^ in 17 patients with 21 nerve injuries in the hand with similar outcomes to standard treatment [54]. The Neurolac^®^ conduit incites an inflammatory foreign body reaction that negated any benefit in rats, but also has been demonstrated in humans [55,56]. In a larger series of 23 patients with 28 nerve injuries in the hand, there were two patients with fistulization of the conduit into the joints [57].

Synthetic conduits are limited by the lack of an internal architecture. A fibrin clot has to form which serves as the scaffold for Schwann cells to grow along and for axonal regeneration to occur on. Fibrin clot instability limits how long a gap can be for axonal regeneration to occur given the lack of an internal architecture in a conduit. Different gap lengths have been investigated in pre-clinical primate models. Regeneration across collagen conduits of 5 cm has been shown to occur, however the results are inferior to outcomes across a 2 cm gap [60,61]. In Krarup et al.’s study, two out of seven median nerve gaps of 5 cm did not have any regeneration across the collagen conduit [61]. As such, synthetic conduits for clinical use are limited to gaps of <3 cm [40,62].

## 4. Regulating Peripheral Nerve Regeneration

Neurotrophic factors are proteins necessary for the function, viability, and regeneration of a neuron after injury [15,34,63,64]. The neurotrophin family includes nerve growth factor (NGF), brain-derived neurotrophic factor (BDNF), glial cell line-derived neurotrophic factor (GDNF), ciliary neuronotrophic factor (CNTF), platelet-derived growth factor (PDGF), vascular endothelial growth factor (VEGF), and many other growth factors [34]. These factors bind to receptors such as the transmembrane tyrosine kinase receptors on the regenerating neuron or the p75 receptor expressed by Schwann cells [34,65,66]. Different neurotrophic factors encourage different aspects of peripheral nerve regeneration. Neurotrophin-4 appears to be important in postnatal development, whereas BDNF appears to have a predominant role after nerve injury [65]. Schwann cell-derived BDNF acts as a retrograde signal from the distal nerve stump to trkB receptors expressed on the regenerating proximal axon to promote axonal elongation [65,67]. NGF attracts regenerating sensory axons within a concentration dependent gradient and enhances Schwann cell motility [68,69,70]. GDNF is a chemoattractant for Schwann cells and stimulates their motility [68,70].

Much progress has been made in elucidating the underlying mechanisms in which neurotrophic factors support nerve regeneration. The transcription factor Sox11 is very important in peripheral nerve regeneration. Sox11 is downregulated in normal adult neurons but will significantly increase expression after injury [71]. Sox11 upregulation promotes neurite elongation, branching, and myelination through activating the regeneration-associated small proline rich protein 1a (Sprr1a) gene [72,73,74]. Sox11 also supports a pro-neuronal survival paradigm through the expression of TNF receptor-associated factor-associated NF-kB activator (TANK) [71,75]. Levels of BDNF are impacted by the transcription factor Sox11 [75,76].

Experimental studies have shown that the Sox11 and its downstream targets are central in peripheral nerve regeneration. Known stimulators of nerve regeneration such as exercise and electrical stimulation lack effects in BDNF or trkB knockout mice [77]. Dorsal root ganglia with Sox11 knockdown have drastically reduced levels of TANK and decreased axonal growth, even in the presence of nerve growth factor [71,75,78]. Inhibition of Sprr1A through the use of siRNA reduces the neuronal stimulation effects of Sox11, highlighting the role of other pathways downstream of Sox11 [73].

The growth factor family of neuregulins also serve an important role in the communication between the regenerating axon and the surrounding Schwann cells. Neuregulin-1 is expressed by the regenerating axon and binds to the ErbB tyrosine kinase receptors on surrounding Schwann cells [79]. Within the Schwann cell, the signal expounds upon multiple pathways including the ERK1/2-MAPK, calcineurin-NFAT, and PI3K-Akt pathways [79]. The broad downstream activity of neuregulin-1 plays a role in Schwann cell growth, motility, and remyelination after injury [80]. Stassart et al. demonstrated that in transgenic mice that overexpressed neuregulin-1, remyelination after injury was restored to its normal thickness [81].

## 5. Exogenous Agents Improving Peripheral Nerve Regeneration

The addition of exogenous neurotrophic factors in peripheral nerve injury repair has garnered attention [82]. Rat tibial nerves reconstructed with fibroblast growth factor within a silicone conduit were found to have 30% more regenerating axons compared to autografts [83]. There is much to be improved regarding growth factor delivery given the short half-life of growth factors [34]. Marquardt et al. utilized scaffolding containing GDNF and tetracycline-inducible GDNF over-expressing Schwann cells to create both the spatial and temporal delivery of GDNF in an animal model, resulting in increased muscle mass [84]. Growth factors are limited by the delivery method and short half-life and do not appear to be promising as a standalone therapy, especially across longer gaps. 

A multitude of agents have been investigated to improve outcomes in peripheral nerve repair. Betamethasone, vitamin E, thyroid hormone, pyrroloquinoline quinone, and erythropoietin have been shown to improve neuronal recovery [85,86,87,88]. Immunophilins have been shown to have an effect on nerve regeneration. Local rapamycin can increase nerve regeneration [89]. FK506, a calcineurin inhibitor, enhances peripheral nerve regeneration and increases axonal sprouting [90,91]. FK506 has a dose dependent benefit, however it also has immunosuppressant properties [92]. Related proteins FK1706 and the FK506-binding protein 52 have similar neurotrophic activity without the immunosuppressive qualities [93,94,95]. These agents have not been widely adopted due to limited demonstrable efficacy in humans and/or side effect profiles.

## 6. Schwann Cell Transplantation in Nerve Regeneration

Creating an acellular nerve allograft requires removing Schwann cells that are otherwise beneficial for nerve regeneration. The addition of exogenous Schwann cells improves peripheral nerve regeneration in acellular nerve grafts and vein conduits in rats [96,97]. Allogeneic Schwann cells may not persist beyond 6 weeks in rats without concurrent immunosuppression [98]. The process of culturing viable Schwann cells is time consuming, however, newer techniques have been developed that shorten the process to 2 weeks [99,100,101]. Levi et al. reported the first human experience of using autologous Schwann cells grown from an injured sciatic nerve and sural nerve graft [102]. The autologous Schwann cells were added to sural nerve grafts to repair a 7.5 cm sciatic nerve defect 30 days after the initial trauma [102]. This case demonstrates the clinical feasibility of harvesting sural nerves for Schwann cell culturing and definitive nerve repair. This method requires harvesting and resultant donor site morbidity. It is, however, a method that involves a procedure already well known to peripheral nerve surgeons without any genetic manipulation.

## 7. Stem Cells in Nerve Regeneration

Stem cells have come to the forefront for their potential benefits in peripheral nerve regeneration. Successful regeneration requires interactions between the neuronal and non-neuronal supporting cells of the extracellular matrix, in addition to neurotrophic factors. Stem cells are of interest given their potential to differentiate into supporting cells to aid in the regenerating process. There are different sources and types of stem cells (See Table 2). Embryonic stem cells are true pluripotent cells that can differentiate into any cell type. Adult stem cells, also known as somatic stem cells, are multipotent and can differentiate into cells along a specific lineage. Takahashi showed that fibroblasts could be reprogrammed into expressing embryonic stem cell markers and behavior by introducing several defined transcription factors [103]. The mechanism of enhanced peripheral nerve repair is not entirely elucidated. Stem cells can differentiate into glial fibrillary acidic protein-positive Schwann cells and can support myelination and the repair process [104]. Stem cells may also differentiate into fibroblast-like cells capable of producing neurotrophic factors in addition to extracellular matrix proteins [105].

The use of stem cells for clinical purposes is subject to various regulations and monitoring. In the United States, this regulatory responsibility falls under the Food and Drug Administration. In 2009, President Barack Obama took a supportive stance for research by lifting a ban that limited federal funding of human embryonic stem cell research to those stem cell lines in use prior to the 2001 ban [106]. At the same time, the National Institutes of Health Guidelines on Human Stem Cell Research were released to stipulate responsible and ethical research of human embryonic and induced pluripotent stem cells [107]. Research is also subject to the individual states’ laws regarding human embryonic stem cells, which can range from fully supportive to restrictive legislation with regard to legalizing or banning embryonic stem cell research and certain practices such as the cloning of human embryos, and provisions for the use of state funding for research [106]. In Europe, the European Medicines Agency classifies cell-based therapies as “advanced therapy medicinal products” [108]. While the European environment is supportive of stem cell research, the infrastructure to allow for the timely transfer of cell-based therapies to the market is still in development [108]. In 2014, Japan enacted the Act on the Safety of Regenerative Medicine and the Revised Pharmaceutical Affairs Law to foster industry collaboration to streamline stem cell-based therapies [109].

### 7.1. Embryonic Stem Cells versus Induced Pluripotent Stem Cells

Embryonic stem cells and induced pluripotent stem cells are similar in differentiation potential. So far induced pluripotent stem cells have shown variable results in the yield of differentiation compared to embryonic stem cells [126].

### 7.2. Embryonic Stem Cells

Embryonic stem cells are self-replicating totipotent stem cells derived from the early human embryo stage, which limits their availability. There are, however, multiple established human embryonic stem cell lines that are available for research. Embryonic stem cells can differentiate into Schwann-like cells that express a Schwann cell phenotype and are shown to associate with axons [110]. Embryonic stem cells injected within an epineurium conduit in rat sciatic nerves demonstrated 64% of normal axonal count versus 7% in autograft across a 1 cm gap [111]. Just over one-third of the embryonic stem cells differentiated to Schwann cells and were shown to survive for at least three months [111]. In another study, human embryonic stem cells were efficiently differentiated to Schwann cells with a yield of 60% [110]. There are, however, ethical concerns with the use of embryonic stem cells. Although differentiation of embryonic stem cells to neural crest stem cells has been performed without the development of tumors, embryonic stem cells have a recognized tendency to form teratomas and immunologic rejection [127,128,129].

### 7.3. Induced Pluripotent Stem Cells

Induced pluripotent stem cells are somatic cells that have been genetically manipulated such that they express a “stem cell-like” phenotype in morphology and growth behavior. Somatic cell induction to stem cells avoids immune rejection and can be taken from sites on the body with lower morbidity. The process of stem cell induction requires cell reprogramming often achieved by retroviral introduction of transcription factors and can take weeks. Induced pluripotent stem cells have been formed from both mouse and human fibroblasts [103,130]. When evaluated in rats, Ikeda demonstrated that nerve conduits coated with induced pluripotent stem cells and fibroblast growth factor had synergistic improvements in regeneration, however the autograft group maintained the best outcomes [112]. Other investigations have shown the induced pluripotent stem cells are similar to embryonic stem cells and, therefore, have a tendency to form teratomas [103,131]. Teratogenicity can be related to the c-Myc gene and has been reduced in protocols using retroviruses without the c-Myc gene, however the process efficiency is reduced [130]. An additional disadvantage is that retroviral silencing occurs when somatic cells make the transformation to the pluripotent state, which affects the stability of induced pluripotent stem cells later in the culturing process [132].

### 7.4. Adipose Stem Cells

Adipose-derived stem cells are promising given that they can be obtained using liposuction techniques without significant morbidity [115]. Wei et al. showed that exogenous adipose-derived stem cells produced similar regenerative outcomes in a rat 10 mm sciatic nerve injury model compared to exogenous Schwann cells within a conduit [114]. These results were improved upon by transducing microRNA-34a into the adipose-derived stem cells in a rat 10 mm sciatic nerve gap model [115]. MicroRNA-34a regulates multiple genes with a resulting pro-neuronal phenotype [133].

Adipose-derived stem cells may promote repair by differentiating into Schwann cells and secreting neurotrophic and angiogenic factors [116]. Pre-clinical studies to date have had variable results using adipose-derived stem cells. Adipose-derived stem cells have questionable durability after differentiating to Schwann-like cells as they are noted to de-differentiate back into stem cells once removed from the differentiation stimulating media [113]. Sowa et al., however, have shown that adipose-derived stem cells may not differentiate to Schwann or Schwann-like cells in-vivo despite in-vitro differentiation [117]. This distinction is important as Tomita et al. showed that undifferentiated adipose stem cells secrete neurotrophic factors, albeit at a lower quantity than adipose stem cells that differentiated into Schwann cells [118]. Thus, the beneficial effects of adipose-derived stem cells are not entirely dependent on in-vivo differentiation to Schwann cells but are enhanced by Schwann cell differentiation.

### 7.5. Neural Crest Stem Cells

Neural crest cell derivatives include peripheral nerves, melanocytes, adrenal glands, teeth, thymus, and Schwann cells [134]. Neural crest cells may be harvested from the skin and have been derived from human induced pluripotent stem cells, fibroblasts, and human embryonic stem cells [128,129,135]. Lin et al. differentiated rat hair follicle neural crest stem cells into neuron and Schwann cells using sonic hedgehog with retinoic acid and neuregulin-1, respectively [119]. The neural crest stem cell derived neurons survived up to 52 weeks post-transplantation with a greater number of regenerated axons in treated groups compared to a xenograft without seeded neurons and Schwann cells [119]. Amoh et al. demonstrated that pluripotent hair follicle stem cells from human scalp could differentiate into Schwann cells and result in greater axonal growth and gastrocnemius muscle contraction in a rat sciatic nerve model [104]. Harvesting hair follicle neural crest stem cells from the skin is favorable given the ease of access with low morbidity and no genetic manipulation required.

To our knowledge, the only reported clinical use of stem cells for peripheral nerve repair was using neural crest stem cells by Grimoldi et al. [120]. Grimoldi et al. used autologous skin-derived stem cells that included neural crest cells to salve an upper extremity in a young woman with polytrauma from a knife stabbing [120]. These stem cells were used within collagen conduits that supported a sural nerve graft across gaps of 8–10 cm [120]. Electromyography and nerve conduction studies demonstrated regeneration across the long gaps, but the motor functional recovery was poor [120]. Her case demonstrates that regeneration across very long gaps can occur with the use of stem cells.

### 7.6. Bone-Marrow Mesenchymal Stem Cells

Bone marrow mesenchymal or stromal cells are a heterogeneous cluster of cells which contain mesenchymal stem cells. These bone marrow derived mesenchymal stem cells can differentiate into Schwann-like cells and improve neural regeneration by expressing trophic factors such as nerve growth factor, BDNF, myelin basic protein, and GDNF [105]. The beneficial effect on nerve regeneration does not appear to be entirely dependent on differentiation into Schwann cells [105]. Cuevas et al. reported the benefits of bone marrow stromal cells in a rat sciatic nerve injury model, however only 5% of the stromal cells differentiated into Schwann cells [121]. In a follow-up experiment, Cuevas et al. showed that sciatic nerve injured rats with transplanted bone marrow stromal cells maintained a significant improvement over control rats at 180 days post procedure [122]. Chen et al. showed the benefit of stromal cells in silicone conduits as measured by the number of axons, reduced muscle atrophy, and walking behavior in rats despite being unable to detect Schwann cells within the silicone conduit [105]. The positive effect on nerve regeneration occurred through the increased production of myelin basic protein and neurotrophic factors [105]. Similarly, Mohammadi et al. showed that undifferentiated bone marrow stromal cells within a vein conduit increased the number and diameter of regenerating myelinated axons and functional improvement to vein grafting alone in a rat sciatic nerve regeneration model [123,124]. When bone marrow stromal cells are differentiated into Schwann-like cells, there is an improvement in rat sciatic nerve regeneration within acellular nerve allografts over undifferentiated bone marrow stromal cells and are overall comparable to the outcomes of autografts [125]. Though bone marrow mesenchymal stem cells promote nerve regeneration, the studies to date show that outcomes are only similar, not improved, when compared to autografts. In the clinical arena, a bone marrow biopsy would be required if autologous transplantation is desired. Further improvement in differentiating bone marrow stromal cells into Schwann cells would enhance the regenerative effect.

## 8. Nerve Tissue Engineering

Nerve tissue engineering refers to the fabrication of biocompatible constructs with or without cellular components that support, encourage, and direct neurite elongation and Schwann cell migration. Neural outgrowth involves close interactions with Schwann cells and the extracellular matrix. By designing biomaterials with internal features or grooves, neural elongation and Schwann cell migration can be guided while minimizing significant fibrosis. Nanofibrous scaffolds are favorable as they have microscopic fibrous features that resemble the microscopic appearance of the extracellular matrix [136]. Yang et al. showed that neural stem cells are capable of attaching to and growing along poly(l-lactic acid) scaffolds [137]. Prabhakaran et al. were able to demonstrate in-vitro mesenchymal stem cell differentiation into neuronal cells capable of expressing neurofilaments on a similar nanofibrous scaffold [138].

An additional challenge will be regeneration across bifurcations of mixed motor and sensory nerves. Romero et al. used sensory specific neurotrophins, and targeted regeneration down a sensory pathway was achieved in-vitro across a “Y” construct. The addition of 3D printing technology is promising as it is possible to create “smart” scaffolding with internal cues and spatial gradients of growth factors to direct nerve regeneration in mixed nerves and across bifurcations [68]. 3D printing technology has been combined with computed microtomography (microCT) to both demonstrate and replicate the internal structure of acellular nerve allografts [139]. It is quite foreseeable to have a peripheral nerve replacement that spans across a bifurcation with a native internal structure for directed regeneration.

## 9. Future Directions

It is well recognized that there is significant room for improvement in peripheral nerve regeneration outcomes. We do not yet have an “off-the-shelf” product for repair of large peripheral nerve defects. The ideal product will be readily available and primed with a protein or cellular substrate to promote regeneration without deleterious immune responses or teratogenicity. In complex injuries involving mixed nerves at a bifurcation, the ideal product will help direct appropriate regeneration.

To our knowledge there has not been a study directly comparing different methods of using exogenous cells, whether they be cultured Schwann cells, or Schwann-like cells from stem cells. As preclinical trials commonly use rat models, it is difficult to simulate large nerve gap defects that are seen by today’s peripheral nerve surgeon.

The sheer time needed for axonal regeneration across large gaps >5 cm favors methods that will utilize Schwann or Schwann-like cells with durable viability. Stem cells from an optimal source are part of the picture. The translational work of Levi and Grimoldi are landmarks in peripheral nerve repair [102,120]. Barriers, however, include the need for clinical trials and the resources needed by a hospital to culture autologous Schwann or Schwann-like cells. The continually improving culturing methods with shorter durations will facilitate both.

## 10. Conclusions

Peripheral nerve surgeons are in need of a technique that consistently produces favorable results for patients with peripheral nerve injuries spanning long gaps. Current modalities under way to improve outcomes include immunomodulation, increasing neurotrophic factors, exogenous Schwann or Schwann-like cells, and advanced nerve scaffoldings. By combining methods to create multimodal techniques, the most significant improvements in peripheral nerve regeneration will be seen.

## Figures and Tables

**Table 1:** Food and drug administration (FDA) approved nerve graft conduits.

Product	Material	Company	Clinical or Preclinical	Comment
NeuraGen^®^	Collagen Type I	Integra LifeSciences Co., Plainsboro, NJ, USA	Taras et al. ^†^ [47] Wangensteen et al. ^†^ [48] Ashley et al. ^†^ [49] Whitlock et al. [46]	Absorbable. Good clinical data for sensory and motor recovery.
NeuraWrap™	Collagen Type I	Integra LifeSciences Co., Plainsboro, NJ, USA	n/a	Protective wrap.
NeuroFlex™	Collagen Type I	Collagen Matrix, Inc., Franklin Lakes, NJ, USA	n/a	Flexible. For gaps 2.5 cm or less.
NeuroMatrix™	Collagen Type I	Collagen Matrix, Inc., Franklin Lakes, NJ, USA	n/a	For gaps 2.5 cm or less.
NeuroMend™	Collagen Type I	Collagen Matrix, Inc., Franklin Lakes, NJ, USA	n/a	Self curling protective wrap.
NeuroTube^®^	Polyglycolic acid	Synovis Micro Companies Alliance, Birmingham, AL, USA	Battiston et al. ^†^ [39] Rosson et al. ^†^ [51] Duncan et al. ^†^ [52]	Absorbable. Acidic degradation limits quantity used [53] Clinical data available for sensory and motor recovery. Risk of extrusion.
Neurolac^®^	Poly(d,l-lactide-*co*-ε-caprolactone)	Polyganics BV, Groningen, The Netherlands	Bertleff et al. ^†^ [54] Meek et al. [55] Hernandez et al ^†^ [56] Chiriac et al. ^†^ [57]	Absorbable. For gaps less than 20 mm. Risk of foreign body reaction.
Salutunnel™	Polyvinyl alcohol hydrogel	Salumedica LLC, Atlanta, GA, USA	n/a	Non-degradable.
AxoGuard™ Nerve Connector	Porcine intestinal submucosa	Cook Biotech, Inc., West Lafayette, IN, USA	n/a	For gaps less than 5 mm.

^†^ denotes clinical study.

**Table 2:** Stem cell sources for peripheral nerve regeneration.

Stem Cell by Source	Advantage	Disadvantages	Clinical or Preclinical Data	Mechanisms of Regenerative Benefit
Embryonic	Totipotent.	Regulatory restrictions. Ethical concerns. Risk of teratoma. Limited availability.	Ziegler et al. [110] Cui et al. [111]	Schwann Cells, GDNF, NGF, FGF, BDNF, angiopoietin-1. 35%–60% yield differentiation to Schwann cells [110,111].
iPSC	Inducible from easily obtainable somatic cells.	Genetic manipulation required. Potential risk of teratoma.	Ikeda et al. [112]	Differentiation to Schwann cells.
Adipose	Ease of harvest. Widely available.	Variability of results. Require stimulation for neurotrophic effect [113].	Wei et al. [114] He et al. [115] Kingham et al. [116] Sowa et al. [117] Tomita et al. [118]	Production of neurotrophic and angiogenic factors [116,118].
Neural crest	Ease of harvest from skin and follicle cells. Can be induced from fibroblasts, hESC, iPSC.	-	Lin et al. [119] Amoh et al. [104] Grimoldi et al.^†^ [120]	Differentiate into neurons and SC.
Bone marrow	Familiarity with harvesting.	Bone marrow biopsy required. Heterogeneity of cells.	Cuevas et al. [121,122] Chen et al. [105] Mohammadi et al. [123,124] Fan et al. [125]	Fibroblast-like cell differentiation. Increase production of myelin basic protein, NGF, BDNF, GDNF, CNTF.

^†^ denotes clinical use.
